# Supplementary material for: Studies of salt and stress sensitivity on arterial pressure in renin-b deficient mice
Source: PLoS One. 2021 Jul 28;16(7):e0250807. doi: 10.1371/journal.pone.0250807 (PMC8318244; doi:10.1371/journal.pone.0250807)
Supplement: Attachment — Submitted filename: Ren-b Salt R1 comments.pdf [file pone.0250807.s002.pdf]

Nakagawa and co-authors have satisfactorily addressed most of the points requested. In my view, this manuscript is almost ready for publication. I have two minor points that should be addressed.

It is really important to recognize that the authors included total renin expression in the hypothalamus and RVLM to support their concept. However, the whole concept of Ren-b disinhibiting Ren-a "secreted" leading to hypertension among the initial cohorts studied is not satisfactorily discussed yet. Indeed, the newly generated gene expression findings are not even mentioned in the current discussion, the authors only mentioned a quotation from their previous study. "Similarly, genetic ablation of renin-b resulted in an augmented expression of renin-a in the RVLM, an important node for autonomic control [22]". Please discuss the previous and current findings together, and make a parallel with the BP phenotype discrepancies.

In addition, I would like to suggest the group to perform Ren-a gene expression in future studies in addition to total renin. In my opinion, measuring Ren-a mRNA is way more robust to affirm that Ren-b deletion disinhibits Ren-a. In case Ren-b is deleted and total renin is increased one may speculate Ren-a was disinhibited. However, looking at the results of the present study "unaltered levels of total renin in the RVLM and decreased in the hypothalamus" the whole concept became weaker. What if there is no Ren-b at all in some brain areas and the effect on Ren-a expression is mediated by some of the neurons supposed to have Ren-b that project to that area. I recognize that proving this concept experimentally is very challenging, but I believe that measuring mRNA levels of both isoforms in brain cardiovascular centers, including wildtype mice, would be helpful to increase the field´s knowledge and to test the authors´ hypothesis.

It is still difficult to understand the final opinion of the authors regarding the modulatory role of stress on the cardiovascular phenotype of mice lacking Ren-b. Below, I copied two sentences, one from the abstract and another from discussion, and they seem to contradict each other.

Their last sentence of the abstract

"These studies suggest that mechanisms unrelated to salt and acute stress alter the cardiovascular phenotype in mice lacking renin-b."

A newly added sentence in the discussion, line 351

"Given the lack of the phenotype that is observed in the current cohorts of renin-b knockout mice, we hypothesized that ablation of renin-b leads to an enhanced susceptibility to hypertension-eliciting psychological and physical stressors."
