# Supplementary material for: Studies of salt and stress sensitivity on arterial pressure in renin-b deficient mice
Source: PLoS One. 2021 Jul 28;16(7):e0250807. doi: 10.1371/journal.pone.0250807 (PMC8318244; doi:10.1371/journal.pone.0250807)
Supplement: Attachment — Submitted filename: Reviewer comments-PLos One-Ren-b Salt.docx [file pone.0250807.s001.docx]

**Response to Reviewers and Editors**

We thank the reviewers and editors for their critique of our work and for the opportunity to address the concerns raised by them. Please note that major changes in the manuscript are denoted by yellow highlighting. We also made all changes required by the style checklist.

**Reviewer #1:**

*1) Nakagawa et al. come up with a very peculiar concept, i.e., that renin-b suppresses renin-a. What would be the logic of having such a system, and how exactly does this happen? The body has many ways to up- or downregulate renin, including BP, salt and Ang II. Since renin-b remains in the cell, how does it affect renin-a? And then apparently this only applies to cells which generate both renin-a and renin-b? Do we have evidence that both occur in the same cell.*

Response: We appreciate your interest in our conceptually innovative hypothesis. Please note that this hypothesis is not new to this paper but is based on our previous publications using these mice. Although we do not understand the precise molecular mechanism, we know that some of the physiological alterations occurring in response to ren-b deletion can be mitigated by suppressing the brain RAS. Since the RAS exerts powerful actions, we agree there must be several ways (including Ren-b) the body regulates its activity. It is still unclear how ren-b suppress ren-a and whether both isoforms are expressed in the same cell or if an inhibitory neuron expressing ren-b suppresses a second order neuron expressing ren-a. We previously authored a comprehensive review article where we proposed possible mechanisms (PMID: 28559391).

*2) The authors hypothesize that mice lacking renin-b would be more responsive to an acute environmental stressor as consequence of brain RAS disinhibition. In the absence of renin-b, the ‘’disinhibition’’ must already be absent, i.e., renin-a is permanently upregulated? Do they have evidence for this in their various cohorts, i.e., might the different response relate to different upregulations of renin-a? And if so, why do we only see the consequence of this during acute stress? This implies that without stress the upregulated renin-a is still suppressed: by what? What then exactly happens during acute stress (mechanistically)? I fail to understand this.*

Response: Evidence suggests that Ren-a is suppressed by Ren-b, but this does not necessarily imply that in absence of Ren-b, the brain RAS is permanently upregulated. In fact, the rationale of this study is that Ren-a is normally dormant, and that the manifestation of a hypertensive phenotype requires not only ablation of Ren-b but an additional exogenous stimulus triggering Ren-a upregulation in the brain. Indeed, injection of angiotensin receptor blockers into the brain of normal normotensive and unstressed mice has little effect on blood pressure suggesting the system is largely dormant under control conditions. As shown in figure 2, different cohort of Ren-b KO mice exhibited a variable degree of blood pressure elevation. Given that these cohorts were housed in different environments we hypothesized that the Ren-b KO mice exhibiting a prominent phenotype must have been exposed to a higher degree of stress, which subsequently might trigger not only upregulation of Ren-a, but also other unknown Ren-b downstream targets. Our data do not support that the stimuli tested here influence the phenotype in mice lacking Ren-b. We have data indicating that Ren-a might not be upregulated in the cohorts exhibiting normal blood pressure. This can be interpreted as Ren-a might be suppressed by other mechanisms but also that a Ren-a was induced.

*3) What about the consequence of renin-b disappearance in the adrenal in the renin-b Null mice? Does it upregulate renin-a at that site? Please provide such data in your model. The renin-b Null mice display proteinuria: why? This occurs in the absence of hypertension. Ren-b Null mice also exhibited an elevated heart rate at baseline, although this is very hard to see in Figure 5. What kind of test was used to reach this conclusion, particularly if it applies only to a few data points?*

Response: We have previously shown that Ren-b KO mice exhibit normal aldosterone levels at baseline but a higher aldosterone secretion when infused with slow-dose Ang II (PMID: 32507043). At your request, we measured renin-a in the adrenal glands and found no significant changes in this cohort of mice (Figure 1). We also observed that the level of corticosterone is not altered in normal conditions or 30 minutes post restraint stress.

Ren-b KO mice exhibited higher protein excretion at baseline compared to the wildtype. Since we have no blood pressure recordings in this cohort of mice, we cannot conclude it occurs in presence or absence of hypertension. In Figure 5, we used two-way ANOVA and there was a significant difference in the heart rate among the genotypes. However, it is indeed interesting that like the modest decrease in plasma renin, the proteinuria was preserved despite what would appear to be a lack of hypertension. This is consistent with our previous study suggesting loss of Ren-b causes some level of end organ damage which may be preserved in the most recent cohort.

*4) The authors refer to previous data on renin ‘’showing a trend to decrease’’ after high-salt. I am not sure what this means. At P=0.094 this cannot be stated (nor is it confirmed here), nor does the ‘’trend’’ toward increased heart weight in Table 2. Please refrain from drawing conclusions if P>0.05, and only discuss/show novel data. Figure 7 also shows no difference between WT and renin-b Null mice.*

Response: Thank you for the suggestion. In response to your comment, we have clarified that there was not statistical significance between these groups. We also included the actual p value in the text, so the reader can make their own conclusions.

*5) Taken together, after Figure 1, it is very hard to see what exactly is the difference between the WT and renin-b Null mice. It seems there is no difference after all? The authors refer to a ‘’decrease’’ in plasma renin, which is not apparent/significant however. There are no differences in salt sensitivity, and even the stress response does not seem to be different. They mention a trend towards increase in heart weight, but this too is NS. The authors suggest that the phenotype is ‘’milder’’ (in reality it is absent?), and speculate about genetic drift. After reading and re-reading this paper, I fail to see what the message is. It seems that the phenotype is entirely lacking. Preferably, the authors first provide a plausible mechanism by which all of this might occur: how does renin-b affect renin-a.*

Response: Based on what we learned from our previous studies, we questioned whether ablation of Ren-b might lead to enhanced responses to a high salt diet and/or acute stress. Then, we also hypothesized that the lack of elevated blood pressure in the current cohorts of Ren-b KO mice might be related to the lack of key environmental or dietary factors unmasking this phenotype. Thus, we performed a series of rigorous studies to address whether ablation of Ren-b induces salt sensitivity and intolerance to stress. As the reviewer well interpret, we conclude that a high salt diet neither stress was sufficient to unmask a hypertensive response in the cohorts of Ren-b KO mice exhibiting normal baseline blood pressure herein.

**Reviewer #2**

1) The study Nakagawa et al. starts discussing a controversial blood pressure phenotype found in their previous studies using mice lacking the intracellular renin isoform (Ren-b). The authors suspected the phenotype discrepancy could potentially be related to stressful experimental conditions in previous experiments that would have induced high blood pressure in Ren-b null mice. To test this hypothesis, the impact of low and high salt diets on blood pressure in mice lacking Ren-b was investigated. Additionally, the authors submitted these mice while on low and high salt diets to an acute restraint-stress protocol to evaluate cardiovascular changes. Overall, this study could not show significant cardiovascular changes among Ren-b deleted animals in comparison to controls, but it uses an interesting experimental approach to test cardiovascular physiology in Ren-b null mice. Below are some comments that should help to improve the work.

Response: We thank the reviewer for the honest and well-intended evaluation of our work.

**Major**:

2) At the end of the abstract the following sentence is written “These studies highlight a complex mechanism that masks/unmasks roles for renin-b in cardiovascular physiology”. What is meant by this sentence, because there was no apparent cardiovascular modulatory effect of Ren-b deletion on high salt diet and stress, neither was proven that stress was correlated to the blood pressure discrepancy in previous studies.

Response: We agree the sentence is misleading. Thus, in the new version it reads: “These studies suggest that mechanisms unrelated to salt and acute stress alter the cardiovascular phenotype in mice lacking renin-b.

3) The authors performed retrospective analyses of the light cycle systolic blood pressure and argued the blood pressure differences may be related to the room dimensions and people traffic that were not favorable during the recordings of cohorts 1-4. Looking at the original publication it is evident that only Sys pressure is increased only during daytime in Ren-b null mice. This information should be included in the present study at least in the text. If light cycle Sys pressure was increased, why this parameter was not investigated separately in the present study rather than the daily average. This should be at least mentioned if there is no difference at baseline as well as during the salt diet challenge. Additionally, do the authors have data recorded during the weekend or holidays, when the room was likely empty, as it seems to be in the dark period? Besides, what is the author’s opinion about the final blood pressure phenotype of Ren-b null mice. Are the data from the previous studies invalid?

Response: Thank you for this important suggestion. We analyzed the systolic blood pressure during the light and the dark cycle separately in the current cohort. As illustrated in Figure 2 below, there is no evidence that there is a significant difference between the Ren-b KO and the wildtype during the light nor dark cycles. This is now mentioned in the discussion section. Based on your suggestion, we reanalyzed the data we acquired for the original publication and can confirm the phenotype is maintained even during the weekends when the room was likely empty (Figure 2B). This suggests that the elevation of blood pressure during the light cycle cannot be attributed to the exposure to noise due to elevated traffic in the room and that the elevation of blood pressure in the previous cohort studied in the original article is valid even when it is reanalyzed in different timepoints. Of note, we cannot rule out the influence of the higher level of vibrations and noise in the old room that are unrelated to the traffic within the facility.

4) The authors mentioned that DOCA-salt reduces central Ren-b and increases Ren-a expression, why they did not investigate the mRNA levels in WT brains after high salt diet.

Response: We appreciate the suggestion as that would have been the key mechanistic experiment if we observed any difference in blood pressure.

5) The authors wrote the following sentence in the abstract. “When renin-b deficient mice were exposed to a high salt diet for a longer duration (4 weeks), there was a trend for increased myocardial enlargement in Ren-b null mice when compared with control mice”. Indeed, it looks like there was a trend to increased heart weight. However, an age matched group on a normal salt diet should be included to show the initial cardiac weight, to check if this trend was not independent of salt.

Response: We measured cardiac weight several times in Ren-b KO at baseline conditions on a normal salt diet, and we have never observed any difference without any exogenous stimulus such as Ang II.

6) Why the authors decided to use different time points upon high salt-diet to evaluate BP, stress and morphology? Blood pressure was investigated 1 week after starting the high salt diet, stress 2 weeks and the morphology 4 weeks after.

Response: The time points were defined according to the aim of each study. Our first aim was to investigate whether Ren-b KO mice are sensitive to a high salt diet. For this, there is a tremendous amount of literature that a week on high salt diet is sufficient to diagnose a salt sensitive individual. Then, the stress test was performed in the following week because our goal was to induce an incremental stimulus to these mice. Since end-organ damage might take longer to develop we performed an additional study where the mice were provided with a high salt diet for 4 weeks.

7) Please give a rational reason why not to investigate the blood pressure response to stress using mice on a normal salt diet too, instead of under low and high salt diets, in particular, because the authors suspected stress could have induced elevation in BP in the cohorts 1-4 in which the animals where likely on a normal salt diet. In the discussion it seems like the experiments were performed in animals on a normal salt diet but this is clearly not the case.

Response: We aimed to investigate whether a second stressor in addition to a low or a high salt diet would be sufficient to unmask the expected phenotype. If we assume that the effect of the amount of salt consumed would cause a linear effect on the responses to stress, it is unlikely that a response to stress in a normal salt diet would be different from a low or a high salt diet.

8) In the results it says “Interestingly, an early cohort of Ren-b null mice exhibited higher urinary protein excretion compared with wildtype mice at baseline. The level of protein excretion was maintained in Ren-b null mice after high salt diet. On the contrary, high salt diet increased urinary protein excretion in WT mice.” What is meant by an early cohort of animals? Were those mice hypertensive and, therefore, not from this study?

Response: The studies in the metabolic cages were part of this study but were not performed in the same animals where telemetry devices were implanted. Thus, we cannot determine if the urinary protein levels are correlated to their blood pressure levels. This sentence has been rewritten for clarity.

9) In their discussion the authors mentioned “Similarly, genetic ablation of renin-b resulted in an augmented expression of renin-a in several brain nuclei including the rostral ventrolateral medulla [20]. In the original publication cited, only the RVLM presented significantly increased expression of Ren-a, the other nuclei had a tendency to have increased mRNA. Also, overall brain mRNA level of Ren-a in Ren-b null mice seems not be to be altered. Please be more precise when citing data from previous studies those from the own group.

Response: The mistake has been corrected. Thank you.

10) Plasma renin activity values shown in figure 7, baseline or after stress, are not higher than 20 ng/mL in average in both WT and Ren-b^null^, while animals submitted to a high salt diet in Table 1 WT present 44.8 and Ren-b^Null^ 24.3. However, high salt diet should suppress renin release and peripheral Ang II production, please discuss this surprising result.

Response: These values are not surprising since blood in the animals with a high salt diet was collected under anesthesia while animals in figure 7 were decapitated, thus the renin levels are expected to be lower. These details are in the methods sections and in the figure legends.

**Minor:**

11) All Graphs should be marked with A, B, C… on top, otherwise it is confusing to find the graph referred in the text and/or figure legend. Please standardize it, currently some figure legend refer to A,B,C… while others not.

Response: As requested the figures were corrected. Thanks for the suggestion.

12) Figure 3, top panel: blue dot legend and n are missing.

Response: We corrected this problem.

13) Figure 7 legend has no general description of content as the previous one.

Response: A detailed description of the content of the Figure 7 is included.

14) On page 3 references should be added to the following sentence “Numerous studies have identified neurogenic actions of elevated salt intake”

Response: The references were added.

15) This sentence in page 3 seems to be incomplete: “For instance, elevated dietary sodium intake sensitizes sympathetic neurons located in the in response to a stimulus [9]”.

Response: As requested, this sentence was re-written.

16) In the methodology it is written “A cohort of Ren-b Null mice without telemetry implants was placed in metabolic cages before and after 3 weeks on a high salt diet”, and table 1 legend says “Table 1: Male Wildtype (WT) and Ren-b null mice were housed in metabolic cages to measure food intake, feces weight, water intake, urine volume, and urinary protein excretion at baseline and after 4 weeks on high salt diet (HSD, 4% NaCl)”. What is correct, 3 or 4 weeks after HSD?

Response: The metabolic cage study was performed at 4 weeks on a high salt diet.

17) Table 2 – The legend says, “Body weight (left column) and tissue weights normalized by body weight (right column)”. The body weight is not shown in this table. The left column shows tissue organ weights, correct?

Response: That is correct. Thank you for detecting this mistake.

18) Methodology of Experiment 2 – please provide more information regarding this experiment, particularly the exact tonicity of the injected solutions, the time course of the experiment and how the urine was sampled.

Response: A more detailed information now is included in the new version.

**Reviewer #3**

The authors compared the salt- and stress- sensitivity of renin-b null and wild type mice with respect to blood pressure, heart rate and renal and metabolic parameters. Their hypothesis was, that under salt- or restrain-stress renin-b null mice may have elevated blood pressure and/or heart rates.

The rational to perform the present study was as follows: The authors previous showed that the genetic removal of renin-b triggered a mild increase in blood pressure and sympathetic nerve activity. The authors previously hypothesized that renin-b maintains brain RAS activity at normal physiological levels by suppressing the classical renin isoform and thus, renin-b deletion would des-inhibit a brain RAS. However, in later studies with other cohorts of renin-b null and wild type mice differences in blood pressure or heart rate were not observed any more. The authors hoped to attribute these discrepancies to differences responses to salt intake and/or stress.

The experiments were very well designed and performed. From the methodological point of view, I have only minor criticism (see below).

However, with respect to the original hypothesis (renin-b inhibits renin-a expression in the brain and des-inhibition of renin-a in the absence of renin-b increases blood pressure) it would be essential to measure renin-a expression again in the brain (or in specific brain areas), i. e. to demonstrate that renin- a mRNA it is still elevated (or not). Only thus the original hypothesis can be supported or must be discarded.

Response: Thank you for the detailed revision of our studies and the accurate description of our conclusions. As you well introduced above there is a discrepancy in the phenotype of the mice studied herein when it is compared with the previous cohorts showing elevated blood pressure. As you know genetic drifts occurs relatively frequently in experimental animal models. We are confident that the data from previous animals were rigorously collected, but for a still unknown reason the model shifted to a milder phenotype. At your request we measured renin levels in hypothalamic samples collected from mice lacking the hypertensive phenotype. Of note, they were fed a normal salt diet. Contrary to the elevated total renin level observed in the Ren-b^Null^ mice that exhibit elevated blood pressure, we found a no increase in the renin mRNA levels in the RVLM. However, there was a significant decrease in renin expression in the hypothalamus (Figure 3). We are certainly positive that the elevated blood pressure in the old cohort can be attributed to the elevated RAS activity in the brain since 1) the expression of renin and other RAS genes are overexpressed in the brain, and 2) the pharmacological blockade of the brain RAS completely blunted the phenotype. Thus, it is possible that a genetic drift or an epigenetic regulation affecting the constitutive expression of the classical renin might partially explain the conflicting observations in the new cohorts of this model.

**Minor comments:**

I would not rely on „trends”( heart weight/BW; plasma renin levels) – moreover, a p-value around 0,09 may not even be considered as a trend.

Response: We agree with the reviewer that p value that is just over the arbitrary threshold of 0.05 gives a low confidence to suggest a difference. In the new version, we state that there are not statistical differences in the heart weight and the plasma renin.

Basal urinary protein excretion was significantly higher in renin-b null – with no further increase under HSD. Please explain possible reasons.

Response: Although we do not have blood pressure recordings in these mice housed in metabolic cages for urine collection, we observed a normal blood pressure in a cohort that was studied just months after. Thus, whether the elevated proteinuria in Ren-b^Null^ mice on a normal salt diet was associated with hypertension is unclear.

Table 2: please add p-values Heart rate

Response: Since we now concluded that there is no difference between the genotypes, it would be misleading to indicate the p value in the table 2.

Fig. 5: the basal heart rates were initially not different (minutes -10 to -5). This was followed by a transient increase, but just before restrain-stress there was again no difference when compared to wild type.

Response: That is correct. The heart rate was only increased during the transition when the animal is transferred from the home cage to the restrainer.
