# Supplementary material for: Studies of salt and stress sensitivity on arterial pressure in renin-b deficient mice
Source: PLoS One. 2021 Jul 28;16(7):e0250807. doi: 10.1371/journal.pone.0250807 (PMC8318244; doi:10.1371/journal.pone.0250807)
Supplement: Attachment — Submitted filename: Reviewer comments-PLos One-Ren-b Salt-resubmission2.docx [file pone.0250807.s003.docx]

**Response to Reviewers and Editors**

We thank the reviewers and editors for their critique of our work and for the opportunity to address the concerns raised by them. Please note that major changes in the manuscript are denoted by yellow highlighting. We also made all changes required by the style checklist.

**Reviewer #1:**

*1) Nakagawa and co-authors have satisfactorily addressed most of the points requested. In my view, this manuscript is almost ready for publication. I have two minor points that should be addressed. It is really important to recognize that the authors included total renin expression in the hypothalamus and RVLM to support their concept. However, the whole concept of Ren-b disinhibiting Ren-a “secreted” leading to hypertension among the initial cohorts studied is not satisfactorily discussed yet. Indeed, the newly generated gene expression findings are not even mentioned in the current discussion, the authors only mentioned a quotation from their previous study. “Similarly, genetic ablation of renin-b resulted in an augmented expression of renin-a in the RVLM, an important node for autonomic control [22]”. Please discuss the previous and current findings together, and make a parallel with the BP phenotype discrepancies.*

Response: Thank you for recognizing our efforts to address your inquiries. Our goal is to provide the most accurate and rigorous data of this new model lacking renin-b, which exhibited elevated blood pressure and elevated total renin in the RVLM in previous cohorts. Surprisingly, these new cohorts not only exhibited normal total renin in the RVLM, but also a paradoxical decrease in renin expression in the whole hypothalamus. We cannot compare the hypothalamic values since we did not measure it in the previous cohorts. However, that the new cohorts failed to increase total renin in the RVLM may indicate that the underlying factor contributing to the phenotype variability is related to alterations in gene regulation in specific cell types. In response to the reviewers’ request, we have added this to the discussion.

We do not have a certain explanation of what and how environmental or epigenetic factors might have caused these changes in the expression of renin in the RVLM. Nevertheless, we believe that this study as well as the previous one has been performed with the highest standards, and thus reporting these new findings would not be detrimental but beneficial to the scientific community.

*2) In addition, I would like to suggest the group to perform Ren-a gene expression in future studies in addition to total renin. In my opinion, measuring Ren-a mRNA is way more robust to affirm that Ren-b deletion disinhibits Ren-a. In case Ren-b is deleted and total renin is increased one may speculate Ren-a was disinhibited. However, looking at the results of the present study “unaltered levels of total renin in the RVLM and decreased in the hypothalamus” the whole concept became weaker. What if there is no Ren-b at all in some brain areas and the effect on Ren-a expression is mediated by some of the neurons supposed to have Ren-b that project to that area. I recognize that proving this concept experimentally is very challenging, but I believe that measuring mRNA levels of both isoforms in brain cardiovascular centers, including wildtype mice, would be helpful to increase the field´s knowledge and to test the authors´ hypothesis.*

Response: Thank you for the suggestion. We agree that it would be useful to specifically measure Ren-a gene expression in our future studies. We agree that it is unclear if both isoforms are expressed in the same cell or if a neuron expressing ren-b modulate a second order neuron expressing ren-a in a different brain region. This question is certainly challenging to address as we have tried to design in situ hybridization probes to distinguish Ren-a vs Ren-b mRNA but none of these have worked thus far. This in part due to the small size of these exons (124 bp and 65 bp) that makes it challenging to design optimal probes and primers. We believe that future studies using single cell RNA sequencing or novel transgenic mice carrying renin-a or renin-b reporter constructs might be helpful.

*3) It is still difficult to understand the final opinion of the authors regarding the modulatory role of stress on the cardiovascular phenotype of mice lacking Ren-b. Below, I copied two sentences, one from the abstract and another from discussion, and they seem to contradict each other. Their last sentence of the abstract: “These studies suggest that mechanisms unrelated to salt and acute stress alter the cardiovascular phenotype in mice lacking renin-b.” A newly added sentence in the discussion, line 351 “Given the lack of the phenotype that is observed in the current cohorts of renin-b knockout mice, we hypothesized that ablation of renin-b leads to an enhanced susceptibility to hypertension-eliciting psychological and physical stressors.”*

Response: We do not see this as a contradiction. The first sentence is an affirmatory sentence where we concluded that these mice are not susceptible to salt and stress, while in the second sentence is not a conclusion but the aim and rationale of our study.

*4) All questions have been addressed, and the authors provided novel data on renin a expression in the adrenal. Please add these to the MS, and discuss them accordingly. These are highly relevant, as their concept is that renin b somehow suppresses renin a. Apparently, this is not true in the adrenal? Why would this only happen in the brain?*

Response: Thank you for the suggestion. In response to your comment, we have included these data to the manuscript. Our interpretation is that there might be differences in how renin-b regulates renin-a in different cells and organs, and this also might be influenced by different physiological and pathophysiological conditions.

**Reviewer #3**

1) My comments have been addressed. The lack of change in renin (renin-a) expression fits to the lack of change in blood pressure. However, there was a decrease - not increase of renin (a) in the hypothalamus. This contradicts the authors conclusion that renin-b decreases renin-a expression. Furthermore, if renin-a in the hypothalamus has any role at all, this decrease should have some consequences. This need to be stated and discussed.

Response: We thank the reviewer for the comment. As discussed in Reviewer #1’s first comment, upregulation of renin-a occurred specifically in the RVLM in the previous, but not in the current cohort of Ren-b KO mice. The expression of renin in the whole hypothalamus were not measured previously, thus it is not comparable. It is possible that there are cell-specific differences in how renin-b regulates renin-a and we might also consider that this regulation might be affected by different physiological and pathophysiological conditions.
